# Conspecific Crop-Weed Introgression Influences Evolution of Weedy Rice (*Oryza sativa* f. *spontanea*) across a Geographical Range

**DOI:** 10.1371/journal.pone.0016189

**Published:** 2011-01-13

**Authors:** Han-Bing Xia, Wei Wang, Hui Xia, Wei Zhao, Bao-Rong Lu

**Affiliations:** Ministry of Education, Key Laboratory for Biodiversity Science and Ecological Engineering, Department of Ecology and Evolutionary Biology, Fudan University, Shanghai, China; University of Umeå, Sweden

## Abstract

**Background:**

Introgression plays an important role in evolution of plant species *via* its influences on genetic diversity and differentiation. Outcrossing determines the level of introgression but little is known about the relationships of outcrossing rates, genetic diversity, and differentiation particularly in a weedy taxon that coexists with its conspecific crop.

**Methodology/Principal Findings:**

Eleven weedy rice (*Oryza sativa* f. *spontanea*) populations from China were analyzed using microsatellite (SSR) fingerprints to study outcrossing rate and its relationship with genetic variability and differentiation. To estimate outcrossing, six highly polymorphic SSR loci were used to analyze >5500 progeny from 216 weedy rice families, applying a mixed mating model; to estimate genetic diversity and differentiation, 22 SSR loci were analyzed based on 301 weedy individuals. Additionally, four weed-crop shared SSR loci were used to estimate the influence of introgression from rice cultivars on weedy rice differentiation. Outcrossing rates varied significantly (0.4∼11.7%) among weedy rice populations showing relatively high overall Nei's genetic diversity (0.635). The observed heterozygosity was significantly correlated with outcrossing rates among populations (*r^2^* = 0.783; *P*<0.001) although no obvious correlation between outcrossing rates and genetic diversity parameters was observed. Allelic introgression from rice cultivars to their coexisting weedy rice was detected. Weedy rice populations demonstrated considerable genetic differentiation that was correlated with their spatial distribution (*r^2^* = 0.734; *P*<0.001), and possibly also influenced by the introgression from rice cultivars.

**Conclusions/Significance:**

Outcrossing rates can significantly affect heterozygosity of populations, which may shape the evolutionary potential of weedy rice. Introgression from the conspecific crop rice can influence the genetic differentiation and possibly evolution of its coexisting weedy rice populations.

## Introduction

Introgression, as defined in its broad sense “the transfer of genes between genetically distinguishable populations” [1], plays an important role in the evolution of plant species [2], [3]. Introgression can promote genetic diversity [4], [5] and affect differentiation of plant populations or species [6], [7]. The introgressed genes that have a natural selective advantage may enhance fitness and adaptability of plant populations occurring in a particular environment [8], [9], stimulating the potential evolution of invasiveness in plants [5]. Currently, the introgression of transgenes from genetically modified (GM) crops to their wild and weedy relatives has aroused worldwide biosafety concerns because such transgene introgression may cause undesired ecological consequences [7], [10], [11]. Therefore, it is particularly important to understand the extent of crop-wild/weed gene flow and introgression that may cause potential evolutionary consequences.

The extent of introgression between species (or populations) relies essentially on their outcrossing ability, which is usually determined by the genetic nature of a plant species [12]–[14]. Species with a greater outcrossing ability will have greater chances to hybridize and introgress between populations, providing that they have a limited reproductive isolation, and *vice versa*. A recent study on modeling of rice gene flow indicated that the outcrossing ability of a pollen-recipient is an important determinant for the level of gene flow [15]. Therefore, estimating outcrossing rates of pollen-recipients will facilitate our understanding on the potential hybridization and introgression of a plant species or population.

Theoretical and empirical studies have indicated evident association between outcrossing and genetic diversity/differentiation of populations [6], [16]–[18]. However, little is known about the relationships among outcrossing rate, genetic diversity, and differentiation of weedy species that coexist with their conspecific crops, from which gene flow and introgression are frequently detected [19]–[21]. Therefore, the conspecific weedy rice (*Oryza sativa* f. *spontanea*; Poaceae) provides an ideal material for studying such relationships, because outcrossing rate, genetic diversity, and differentiation of weedy populations can be estimated relatively easily.

Weedy rice can be defined as any spontaneously and strongly shattering rice that occurs in cultivated rice fields. It is distributed all over the world, where rice is grown. Weedy rice can be discriminated from wild rice species by its habitats because weedy rice only occurs in or nearby rice fields and the wild species in natural habitats. Weedy rice is characterized by its strong seed shattering and dormancy, in addition to a red pericarp, which serves as a key to distinguish weedy rice from cultivated rice. Groups of weedy rice from various parts of the world may have separate evolutionary origins [22], [23]. In regions where wild and cultivated rice coexists, weedy rice may have a hybrid origin involving wild rice [24], [25], while in other regions where no wild rice is found, weedy rice may have a origin only involving cultivated rice [26]–[29]. Weedy rice has been found in many rice planting regions in China. Severe infestation of weedy rice has been reported in NE and E China, probably due to the change of rice farming styles from traditional transplanting to direct seedling (or other no-till cultivation) in the past ten to twenty years [27]. Morphological variation in Chinese weedy rice is relatively small, compared to other weedy rice groups in the world. Because there is no natural distribution of wild rice is in NE and E China, weedy rice found there may have originated from cultivated rice [27].

Weedy rice flowers almost simultaneously in many rice planting regions with its cultivated counterpart occurring in the same fields, and spontaneous hybridization (or gene flow) between cultivated rice and weedy rice is frequently reported [20], [21], [30], [31]. For example in NE and E China, weedy rice flowers simultaneously or slightly earlier than the rice cultivars grown in the same field, resulting frequently spontaneous crop-weed hybridization. As known by many rice scientists that weedy rice has a special feature of mimicking cultivated rice appearing in the same fields after only a few generations [22], [23], [32], which indicates the important role of introgression from cultivated rice to weedy rice in such mimicry. The study of introgression in weedy rice will shed meaningful light on the understanding of its adaptive evolution. In addition, knowledge on introgression of crop genes to weedy rice will also facilitate the risk assessment of ecological impacts caused by transgene flow from GM rice.

Using microsatellite (SSR) fingerprints, we estimated outcrossing rates, genetic diversity, and differentiation of 11 weedy rice populations collected from NE and E China. In addition, we also examined the possible allelic introgression from cultivated rice grown in the same fields. The primary objectives of this study were to (1) determine outcrossing rates and genetic diversity of the 11 weedy rice populations, and the correlation between outcrossing rates and genetic diversity; (2) estimate genetic differentiation among the weedy rice populations across a geographical range; and (3) detect the possible allelic introgression from cultivated rice at different locations that may have affected the differentiation of weedy rice.

## Methods

### Sampling of weedy rice populations and rice varieties

**Figure 1:**
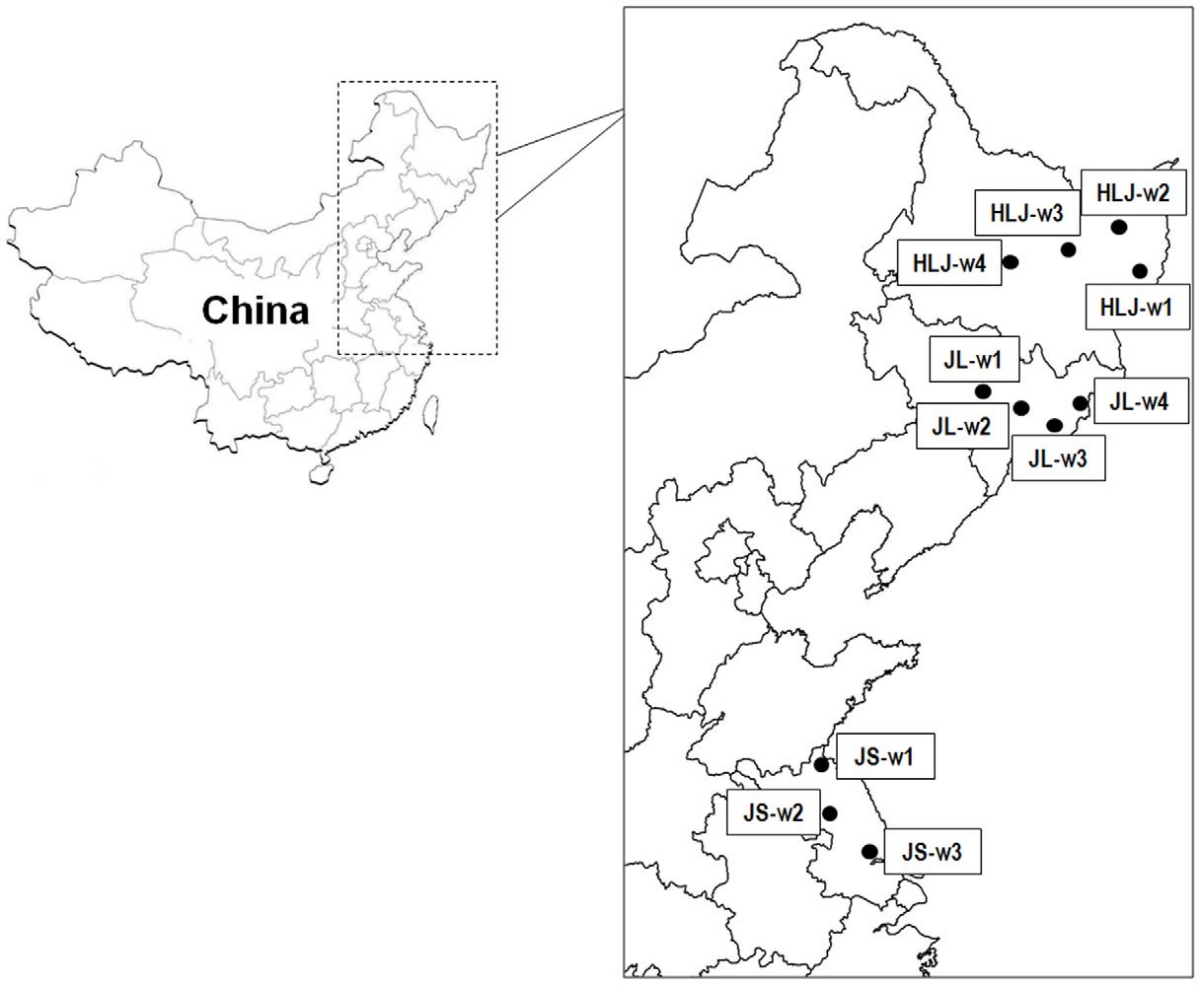
Spatial location of weedy rice populations used for outcrossing and genetic diversity studies. The HLJ-1 to HLJ-4 populations were collected from Heilongjiang Province; the JL-1 to JL-4 populations from Jilin Province; and the JS-1 to JS-4 populations from Jiangsu Province. The detail information of each population refers to Table 1.

For the study of outcrossing and genetic diversity, a total of 11 weedy rice populations occurring in rice fields were sampled from Heilongjiang, Jilin, and Jiangsu Provinces of NE and E China, where modern rice varieties were grown. Spatial locations of the sampled populations were recorded using a global positioning system (GPS) (Table 1), and the spatial distance between weedy rice populations was >50 km (Fig. 1). The weedy rice plants of each population were found to be scattered in a rice field within an area ca. 1000 m^2^. The estimated densities of weedy rice populations varied between 2∼60 plants per 100 m^2^ (Table 1). In Heilongjiang and Jilin Provinces, rice is cultivated only once a year, and after rice harvest, the fields are usually left fallow till the next planting season. In Jiangsu Province, the farming system follows a rice-wheat or rice-oil rape rotation within a year. In general, the environment of the same rice fields where a weedy population occurred was relatively uniform.

**Table 1:** Sampling and location of weedy rice populations and the coexisting rice cultivars from northeast and east China for the estimate of outcrossing rates and genetic diversity.

Code of weedy rice populations	Locality	Longitude	Latitude	Density of weedy rice (plants/100 m^2^)	No. of families (progeny) for outcrossing estimate*	No. of plants for diversity estimate	Name and code of coexisting rice cultivars**
HLJ-w1	Heilongjiang	133°5′28″E	45°56′21″N	30	20 (598)	29	Suijing-3 (HLJ-c1)
HLJ-w2	Heilongjiang	132°4′3″E	47°14′53″N	9	20 (500)	28	Kongyu-131 (HLJ-c2)
HLJ-w3	Heilongjiang	130°43′47″E	46°59′32″N	3	20 (500)	25	Changlixiang (HLJ-c3)
HLJ-w4	Heilongjiang	127°15′14″E	47°11′47″N	2	16 (400)	16	Kongyu-131 (HLJ-c4)
JL-w1	Jilin	127°18′52″E	43°46′11″N	15	20 (500)	24	97-98 (JL-c1)
JL-w2	Jilin	128°18′0″E	43°25′23″N	30	20 (529)	30	Ziwu (JL-c2)
JL-w3	Jilin	128°24′18″E	42°57′18″N	3	20 (500)	30	Honggaoliang (JL-c3)
JL-w4	Jilin	129°28′54″E	43°0′3″N	3	20 (500)	29	CV-1 (JL-c4)
JS-w1	Jiangsu	119°14′42″E	34°34′31″N	60	20 (500)	30	CV-2 (JS-c1)
JS-w2	Jiangsu	119°1′12″E	33°30′26″N	6	20 (500)	30	Huaidao-4 (JS-c2)
JS-w3	Jiangsu	120°0′17″E	32°13′51″N	3	20 (500)	30	CV-3 (JS-c3)

*Numbers in parentheses indicate the numbers of progeny.

**Code is included in parentheses.

To meet our objectives, three independent sets of materials were included in this study. To estimate outcrossing rates, 16∼20 weedy rice plants (as family) were collected from each population, with a spatial distance of >2 m between sampled plants. About 20∼25 mature seeds (as progeny) from each plant were randomly collected, and a total of 400∼600 seeds from each population were sampled. Consequently, a total of 5527 progeny (seeds) from the 11 weedy rice populations were included for analyses (Table 1). To estimate genetic diversity and differentiation, 16∼30 weedy rice plants from each population were randomly collected and a total of 301 plants were included for analyses. In addition, 7∼10 plants of *Japonica* rice cultivars (pure lines) coexisting in the same fields were also sampled from each of the 11 sites, making up a total of 106 rice plants from the sampled sites. One to three panicles from each cultivar and weedy plant were collected and placed separately in a paper seed-bag. Panicles were air dried under natural conditions.

### DNA extraction and microsatellite analysis

Seeds of weedy rice and cultivated rice were germinated in the laboratory of Fudan University at the room temperature (25±3°C) about four months after the samples were collected to release potential seed dormancy. The total genomic DNA was extracted from 10-day-old fresh seedlings following a modified cetyltrimethyl ammonium bromide (CTAB) protocol [33].

SSR primer pairs designed based on published genome sequence of cultivated rice (http://www.gramene.org) were randomly selected and used for analyzing weedy rice populations and rice cultivars. To estimate outcrossing rates and crop specific alleles that were defined as the alleles absent in families (mother plants) but present in progeny (seeds), six highly polymorphic SSR loci were selected, as recommended by Jarne and David [34]. To estimate genetic diversity and population structure, 22 SSR loci were selected in this study In addition, four highly polymorphic SSR loci were selected and used to analyze the population structure of weedy rice populations and rice cultivars (Table 2).

**Table 2:** SSR primer pairs used for DNA amplification with their molecular weight of fragments detected in eleven weedy rice populations.

Primer ID*	Chromosome no.	Forward primer (5′-3′)	Reverse primer (5′-3′)	Molecular weight (bp)
**RM218**	3	TGGTCAAACCAAGGTCCTTC	GACATACATTCTACCCCCGG	120∼150
**RM276**	6	CTCAACGTTGACACCTCGTG	TCCTCCATCGAGCAGTATCA	124∼142
**RM180**	7	CTACATCGGCTTAGGTGTAGCAACACG	ACTTGCTCTACTTGTGGTGAGGGACTG	109∼115
**RM219**	9	CGTCGGATGATGTAAAGCCT	CATATCGGCATTCGCCTG	170∼217
**RM21**	11	ACAGTATTCCGTAGGCACGG	GCTCCATGAGGGTGGTAGAG	131∼157
**RM167**	11	GATCCAGCGTGAGGAACACGT	AGTCCGACCACAAGGTGCGTTGTC	124∼158
RM220	1	GGAAGGTAACTGTTTCCAAC	GAAATGCTTCCCACATGTCT	110∼131
RM24	1	GAAGTGTGATCACTGTAACC	TACAGTGGACGGCGAAGTCG	153∼183
RM208	2	TCTGCAAGCCTTGTCTGATG	TAAGTCGATCATTGTGTGGACC	167∼183
RM263	2	CCCAGGCTAGCTCATGAACC	GCTACGTTTGAGCTACCACG	126∼176
RM55	3	CCGTCGCCGTAGTAGAGAAG	TCCCGGTTATTTTAAGGCG	233∼240
RM241	4	GAGCCAAATAAGATCGCTGA	TGCAAGCAGCAGATTTAGTG	127∼139
RM280	4	ACACGATCCACTTTGCGC	TGTGTCTTGAGCAGCCAGG	146∼169
RM249	5	GGCGTAAAGGTTTTGCATGT	ATGATGCCATGAAGGTCAGC	127∼157
RM289	5	TTCCATGGCACACAAGCC	CTGTGCACGAACTTCCAAAG	103∼109
RM253	6	TCCTTCAAGAGTGCAAAACC	GCATTGTCATGTCGAAGCC	129∼140
RM11	7	TCTCCTCTTCCCCCGATC	ATAGCGGGCGAGGCTTAG	122∼140
RM234	7	ACAGTATCCAAGGCCCTGG	CACGTGAGACAAAGACGGAG	109∼154
RM44	8	ACGGGCAATCCGAACAACC	TCGGGAAAACCTACCCTACC	112∼136
RM215	9	CAAAATGGAGCAGCAAGAGC	TGAGCACCTCCTTCTCTGTAG	147∼157
RM228	10	CTGGCCATTAGTCCTTGG	GCTTGCGGCTCTGCTTAC	112∼160
RM258	10	TGCTGTATGTAGCTCGCACC	TGGCCTTTAAAGCTGTCGC	122∼148
RM202	11	CAGATTGGAGATGAAGTCCTCC	CCAGCAAGCATGTCAATGTA	166∼182
RM235	12	AGAAGCTAGGGCTAACGAAC	TCACCTGGTCAGCCTCTTTC	102∼144

*Primer pairs with underlines were used in diversity analysis; primer pairs with **Bold** letters were used in outcrossing of weedy rice and crop-specific allele analysis; primer pairs with underlined **Bold** letters were used for cluster analysis of weedy and cultivated rice.

Polymerase chain reactions (PCR) were carried out in a volume of 20 µL containing 1× buffer, 1 mM each of dATP, dCTP, dGTP and dTTP, 10 mM of SSR primer, 50 ng of genomic DNA and 0.6 unit of Taq polymerase [27]. The reactions were performed in a PTC 10096v thermocycler (MJ Research Inc., Watertown, MA, USA) with 4 min initial denaturation at 94°C, 36 cycles of 40 s at 94°C, 30 s at 55°C and 40 s at 72°C, followed by a final extension of 10 min at 72°C. The PCR products were mixed with 8 µL SSR loading buffer (98% formamide, 0.25% xylene cyanol FF, 0.25% bromophenol blue, 10 mmol EDTA pH 8.0) before loading on 6% polyacrylamide denaturing gel (20 cm long, 12.5 cm wide and 1 mm thick). The pUC19 DNA/MspI (HpaII) marker ladder (Fermentas International Inc) was also loaded to determine molecular size of amplified alleles. Electrophoresis was conducted at the voltage of 400 V for about 3 hours to separate DNA fragments according to different base pairs (bp). Electrophoretic products were revealed using the modified silver staining procedure [35].

### Data scoring and analysis

#### Data scoring

Given the co-dominant nature of SSR markers, the amplified DNA fragments of a given locus were scored as genotype data for each weedy rice individual. For outcrossing analyses, genotype data of progeny was recorded as homozygous genotypes (AA, BB, CC…) or heterozygous genotypes (AB, AC, BC…) and formatted according to the MLTR requirement to be used for outcrossing rates estimation under MLTR model [36]. For the analyses of genetic diversity and differentiation, genotype data were recorded according to the molecular weight (bp) of SSR alleles, either as homozygous genotypes (120/120, 140/140, 150/150…) or heterozygous genotypes (120/140, 120/150, 140/150…), based on migrating rates of the marker ladder [pUC19 DNA/MspI (HpaII)] on electrophoretic gels.

#### Parameters of outcrossing and genetic diversity

The mating system of weedy rice populations was calculated from 5527 progeny using a multilocus mixed mating model [36], based on six highly polymorphic SSR loci. The following parameters were calculated: (1) multilocus outcrossing rate (*t_m_*); (2) single-locus outcrossing rate (*t_s_*); (3) the proportion of biparental inbreeding to the total outcrossing rate [(*t_m_*-*t_s_*)/*t_m_*] [37]; (4) the number of pollen donor plants (effective paternity: *Pa*) as estimated by inverse of multilocus paternity correlation [38]; (5) population substructure (*Subs*) estimated by the differences between correlation of pollen genotypes calculated by multilocus and single-locus [36]. The Newton–Raphson method was used to solve the likelihood equation for estimating the maximum likelihood, as recommended by Ritland [36]. The standard deviation (s.d.) of the parameters was calculated based on 1000 bootstraps, where the sampling units were the progeny within a family. All parameters relating to mating system were calculated by a software MLTR ver. 3.0 [36].

The parameter of inbreeding coefficient (*f*) was calculated to estimate whether the observed outcrossing rates of weedy rice populations based on SSR markers and MLTR model deviated from theoretically expected outcrossing rates under inbreeding equilibrium (IE). Inbreeding coefficient (*f*) was obtained as follows: (1) calculating the most possible maternal genotypes observed in each weedy rice population based on the MLTR model; (2) calculating inbreeding coefficient (*f_M_*) of maternal plants in each population based on maternal genotypes by the formula: *f_M_*  = 1 - *H_o_*/*H_e_*, where *H_o_* and *H_e_* indicated the observed heterozygosity and expected heterozygosity, respectively; (3) calculating the expected inbreeding coefficient (*f_eq_*) in each population by the formula: *f_eq_*  =  (1 - *t_m_*)/(1+*t_m_*) [6]. A *t*-test of paired samples was performed to detect the significant differences between the observed (*f_M_*) and expected (*f_eq_*) coefficients.

Genetic diversity was estimated from 301 plants of 11 weedy rice populations based on 22 SSR loci using the MSA (microsatellite analyzer) software ver. 4.05 [39]. The following diversity parameters were calculated: (1) Wright's inbreeding coefficient (*F_is_*) [40]; (2) the average number of alleles per locus (*A*); (3) the percentage of polymorphic loci (*P*); (4) Nei's genetic diversity (*GD*) [41]; and (5) the average of observed heterozygosity (*H_o_*). Private alleles of each weedy rice population were analyzed using the GDA (genetic data analysis) software ver. 1.1 [42].

Correlations between obtained outcrossing rates from mating estimation and genetic diversity parameters (*A*, *GD*, and *H_o_*) were analyzed using the Pearson's correlation method packaged in the SPSS software ver. 12.0 (http://www.spss.com/). The correlation was illustrated by a linear model (y = a+ bx) using the STATISTICA ver. 6.0 (StatSoft Inc., OK, USA).

#### Differentiation of populations

Nei's unbiased genetic distances [41] were calculated using the MSA software ver. 4.05 [39] based on the SSR data matrix generated from 301 plants of 11 weedy rice populations. A UPGMA (unweighted pair group method with arithmetic averages) tree was constructed based on the cluster analysis of genetic distances, using the software NTSYS ver. 2.02 [43]. To determine the statistical support at branching points, 1000 trees were constructed from bootstrap re-samplings of data, and the number of times that a clade appeared in 1000 bootstrap sample was evaluated using CONSENSE program packaged in PHYLIP software ver. 3.66 [44].

The Mantel test [45] was conducted to determine the correlation between genetic distance and geographical distance of weedy rice populations, using the ARLEQUIN software ver. 3 [46]. The significance of Mantel test was estimated with 1000 permutations.

#### Relationship between weedy and cultivated rice

To estimate the introgression of crop alleles to weedy rice, the paternity of progeny in weedy rice was determined. The following procedures were taken to achieve this: (1) estimating frequency of alleles from paternal and maternal plants by MLTR software; (2) determining paternal specific alleles by comparing alleles from both parents, alleles that only appear in pollen donors were defined as paternal specific allele; (3) determining the paternal specific alleles that could only come from the crop by examining alleles in cultivated rice coexisting in the same fields, as crop specific alleles.

Nei's unbiased genetic distances of 11 weedy rice populations (301 plants) and rice varieties (106 plants) that coexisted in the same fields were calculated together based on four shared SSR primer pairs (RM21, RM218, RM219, RM276) that highly represented genetic diversity and structure of the crop and weed, using the MSA software ver. 4.05. An UPGMA tree of weedy and cultivated rice was constructed based on the cluster analysis of Nei's genetic distances, using the software NTSYS ver. 2.02 [43].

## Results

### Outcrossing rates and heterozygosity of weedy rice populations

**Figure 2:**
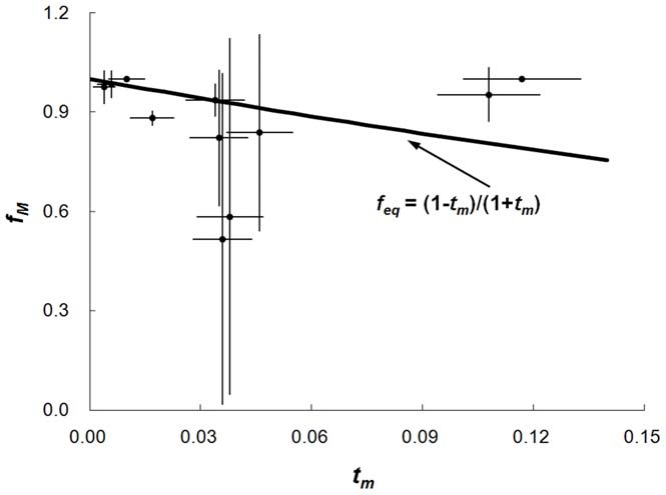
The inbreeding coefficients (*f_M_*) of maternal plants plotted as a function of outcrossing rates (*t_m_*). Bars indicate the standard deviation of the means. Expected inbreeding coefficients at inbreeding equilibrium (*f_eq_*), calculated by (1 - *t_m_*)/(1+*t_m_*), is plotted in a bold line.

Average outcrossing rates of the 11 weedy rice populations were 4.1% as estimated by multilocus outcrossing rate (*t_m_*), and 2.9% by single-locus outcrossing rate (*t_s_*), indicating in general very low frequency of outcrossing in weedy rice populations, which correspond to the self-pollinating nature of weedy rice. A relatively high level of variation in outcrossing rates was found among populations (*t_m_* ranging from 0.4∼11.7% and *t_s_* from 0.3∼8.6%). The lowest multilocus outcrossing rate was found in the JL-4 population and the highest in the HLJ-4 population (Table 3). To examine the reliability of observed outcrossing rates, we conducted the paired-samples *t*-test between maternal inbreeding coefficient (*f_M_*: calculated based on *H_e_*/*H_o_* ratio) and expected inbreeding coefficient (*f_eq_*: calculated based on observed outcrossing rates; see Materials and methods) at inbreeding equilibrium. The obtained results showed no significant deviation (*t* = -1.098; *P* = 0.298) between *f_M_* and *f_eq_*, suggesting in general that the outcrossing rates detected in this study were reliable (Fig. 2). The inbreeding coefficient of most possible maternal populations ranged from 0.516∼1.000 (Fig. 2). A low level of biparental inbreeding that indicated outcrossing among closely related individuals was detected in all weedy rice populations (Table 3).

**Table 3:** Outcrossing rates of eleven weedy rice populations estimated using the multilocus mixed mating model.

Population code	*t_m_*	*t_s_*	(*t_m_* - *t_s_*)/*t_m_*	*Pa*	*Subs*
HLJ-1	0.017 (0.006)	0.013 (0.004)	29.4%	5.00	−1.199 (0.597)
HLJ-2	0.036 (0.008)	0.032 (0.007)	11.1%	5.00	−1.199 (0.620)
HLJ-3	0.035 (0.008)	0.026 (0.006)	25.7%	5.00	−1.178 (0.340)
HLJ-4	0.117 (0.016)	0.079 (0.013)	32.5%	4.65	0.360 (0.142)
JL-1	0.108 (0.014)	0.086 (0.012)	20.4%	2.44	0.350 (0.132)
JL-2	0.038 (0.009)	0.032 (0.008)	18.4%	5.00	0.000 (0.023)
JL-3	0.034 (0.008)	0.012 (0.003)	64.7%	1.64	−0.388 (0.403)
JL-4	0.004 (0.003)	0.003 (0.002)	25.0%	5.00	0.000 (0.639)
JS-1	0.010 (0.005)	0.007 (0.004)	30.0%	1.00	0.000 (0.518)
JS-2	0.006 (0.004)	0.003 (0.002)	50.0%	5.00	0.000 (0.557)
JS-3	0.046 (0.009)	0.021 (0.004)	54.3%	1.11	0.190 (0.388)

*t_m_*: outcrossing rate estimated by multiple loci; *t_s_*: outcrossing rate estimated by single loci; (*t_m_* - *t_s_*_)/_ *t_m_*: the proportion of the biparental inbreeding to the total outcrossing (%); *Pa*: number of pollen donator parents (effective paternity); *Subs*: substructure estimated by difference of multilocus outcrossed paternity correlation and single-locus outcrossed paternity correlation. Numbers in parentheses indicate standard deviations (s.d.).

Average effective paternity (*Pa*), indicating the average number of genetically distinct pollen donors (father plants) involved in hybridization, was nearly four plants in weedy rice populations, with the highest number of five plants and the lowest of one plant (Table 3). In addition, some weedy rice populations (e.g., HLJ-1, HLJ-2, and HLJ-3) showed a weak genetic substructure, as estimated by differences in correlation between paternal genotypes detected in progeny based on multilocus and single-locus measurement. Other weedy rice populations did not show such a substructure (Table 3).

Wright's inbreeding coefficient (*F_is_*) estimated from 22 SSR primer pairs of 301 plants was as high as 0.987 (Table 4), indicating the inbreeding nature of weedy rice with deficient heterozygote in populations, compared to expectations under Hardy-Weinberg equilibrium. Genetic diversity detected in weedy rice populations was relatively high, considering the inbreeding nature of weedy rice. A total of 107 alleles (102∼240 bp) with an average of 4.86 per loci were generated by 22 SSR primer pairs (Tables 2 and 4). An average of ca. 2∼9% private alleles was detected in most of the weedy rice populations. The most polymorphic locus was RM220 (8 alleles) and the least polymorphic locus was RM280 (3 alleles). The percentage of polymorphic loci ranged from 59.1∼100% among populations (Table 4). The overall Nei's genetic diversity (*GD*) of the 11 weedy rice populations was 0.635, with the highest value in the HLJ-4 population (0.453) and the lowest in the JL-2 population (0.145) (Table 4). The observed heterozygosity (*H_o_*) varied considerably among the 11 weedy rice populations, with the highest value of *H_o_* = 0.023 in the HJL-4 and JL-1 populations, and the lowest value as *H_o_* = 0.000 in JL-4, JS-2, and JS-3 populations (Table 4).

**Table 4:** Parameters of genetic diversity in eleven weedy rice populations based on 22 SSR primer pairs.

Population code	*F_is_*	*A*	*P*%	*GD*	*H_o_*
HLJ-1	0.982	2.273 (0.827)	81.8	0.358	0.007 (0.014)
HLJ-2	0.966	2.136 (0.774)	81.8	0.337	0.012 (0.021)
HLJ-3	0.985	2.364 (0.790)	86.4	0.376	0.006 (0.015)
HLJ-4	0.949	2.409 (0.796)	90.9	0.453	0.023 (0.050)
JL-1	0.916	2.046 (0.722)	81.8	0.273	0.023 (0.036)
JL-2	0.927	1.818 (0.795)	59.1	0.145	0.012 (0.026)
JL-3	0.986	2.227 (0.973)	72.7	0.324	0.005 (0.012)
JL-4	1.000	2.091 (0.868)	77.3	0.258	0.000 (0.000)
JS-1	0.989	2.500 (0.740)	95.5	0.429	0.005 (0.012)
JS-2	1.000	2.636 (0.790)	100.0	0.220	0.000 (0.000)
JS-3	1.000	2.000 (0.960)	77.3	0.385	0.000 (0.000)
Overall	0.987	4.864 (1.424)	100.0	0.635	0.007 (0.008)

*F_is_*: Wright's (1978) inbreeding coefficient [40]; *A*: average number of alleles per locus; *P*: percentage of polymorphic loci; *GD*: Nei's genetic diversity [41]; *H_o_*: observed heterozygosity. Numbers in parentheses indicate standard deviations (s.d.).

**Figure 3:**
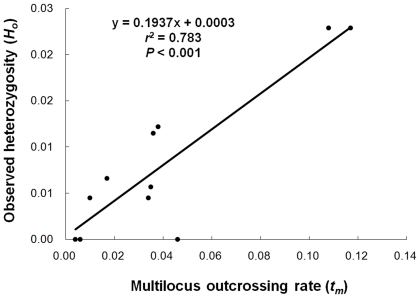
Correlation between outcrossing rates (*t_m_*) and observed heterozygosity (*H_o_*) of weedy rice populations.

Considering the similar variation patterns of outcrossing rates and observed heterozygosity in weedy rice populations, Pearson's correlation between the two groups of values were analyzed. Results showed a significant and positive correlation (for *t_m_*, *r* ^2^ = 0.783; *P*<0.001; for *t_s_*, *r* ^2^ = 0.895; *P*<0.001) between outcrossing rates and observed heterozygosity in the 11 weedy rice populations. The correlation between multilocus outcrossing rates and observed heterozygosity could be well described by a linear model y = 0.1937x+0.0003 (Fig. 3), whereas that between single-locus outcrossing rates and observed heterozygosity as y = 0.2755x+0.0004. However, no significant correlation was detected between multilocus or single locus outcrossing rates and Nei's genetic diversity or average number of alleles per locus (data not shown).

### Genetic differentiation among weedy rice populations

**Figure 4:**
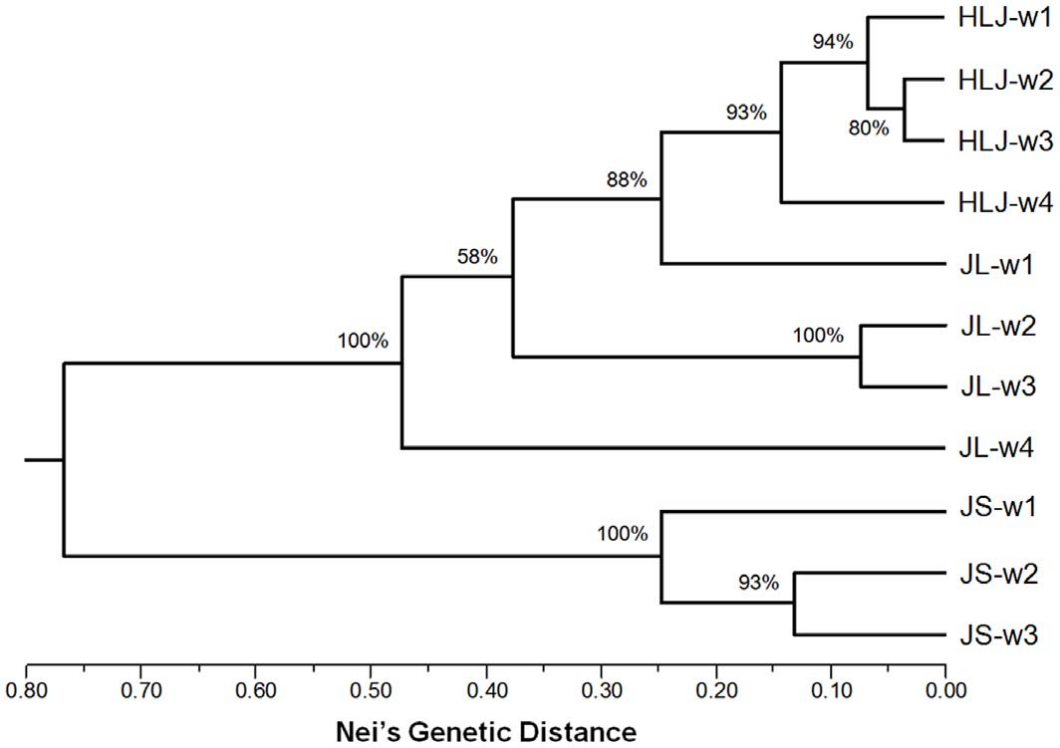
The UPGMA dendrogram of weedy rice populations, based on Nei's unbiased genetic distance. The numbers on the branches indicate the percentages of times a cluster appeared in 1000 bootstrap samples.

Nei's unbiased genetic distance was used to estimate genetic differentiation among weedy rice populations. The pair-wise genetic distances among weedy rice populations varied considerably from 0.036 to 0.874, with an average value of 0.505, indicating a considerable genetic differentiation among weedy rice populations. The UPGMA dendrogram indicated an obvious differentiation pattern among weedy rice populations with a high bootstrap-value support (Fig. 4). The 11 weedy rice populations were clustered into two distinct groups. Three populations from Jiangsu Province were included in one group and other eight populations from Heilongjiang and Jilin Provinces were included in another group. It seemed that weedy rice populations from same regions showed a closer genetic relationship than those from different regions.

The Mantel test indicated a highly significant correlation (*r* ^2^ = 0.734; *P*<0.001) between the genetic distances and spatial distances of weedy rice populations, indicating that genetic differentiation among weedy rice populations was significantly correlated with their geographical distribution. The possible flow of SSR alleles from cultivated rice to coexisting weedy rice populations was examined by analyzing the paternity in weedy rice seedlings (progeny), given that a weedy rice mother plant (family) was always surrounded by cultivated rice plants (see the data of low density of weedy rice in Table 1). Of 165 SSR alleles detected, 28 paternal specific alleles from pollen donors were found in weedy rice progeny in nine weedy rice populations (Table 5), indicating that the weed may have acquired crop specific alleles from cultivated rice. Comparison between the 28 paternal specific alleles and those from weedy rice generated based on the 6 highly polymorphic SSR loci indicated that most of the paternal specific alleles (18 out of 28) were not detected in weedy rice populations of the same field. This result strongly suggested the possibility of paternal specific alleles in weedy rice from the coexisting rice cultivars. Further comparison between the paternal specific alleles calculated based on the paternity of weedy rice progeny and the actual crop alleles detected from the coexisting rice varieties with weedy rice populations indicated some shared alleles (seven) in weedy and cultivated rice (Table 5). Among these shared alleles, four were fixed in local rice varieties, which provided a strong evidence for crop-to-weed introgression. These findings demonstrated that introgression had happened from cultivated rice to coexisting weedy rice populations.

**Table 5:** Paternal specific alleles and their frequencies in weedy rice populations as estimated by MLRT (Ritland, 2002) [36].

Population code	Locus	Paternal allele (frequency)	Allele detected in rice varieties (frequency)
HLJ-1	RM21	F (0.37)	–
	RM219	B (0.18)	–
HLJ-2	RM21	E* (0.18), F (0.12)	E (1.00)
	RM218	C (0.06)	–
HLJ-3	RM21	F (0.37)	–
	RM219	B* (0.25)	B (0.14)
HLJ-4	RM21	B (0.19)	–
	RM218	C (0.36), D (0.04)	–
	RM219	B* (0.26)	B (1.00)
JL-1	RM21	B (0.02), F* (0.19)	F (0.90)
	RM218	C* (0.02)	C (0.10)
	RM276	F* (0.37), D (0.02)	F (1.00)
JL-2	RM276	F* (0.71)	F (1.00)
JL-3	RM167	E (0.23)	–
	RM21	C (0.17)	–
JS-1	RM167	B (0.39)	–
	RM21	A (0.20)	–
	RM218	A (0.39)	–
	RM219	B (0.39), C (0.20)	–
	RM276	F (0.59)	–
JS-3	RM167	B (0.22)	–
	RM21	B (0.04)	–
	RM218	A (0.44)	–

The shared alleles detected in rice varieties grown in the same field were indicated in the last column.

*The crop-specific alleles.

**Figure 5:**
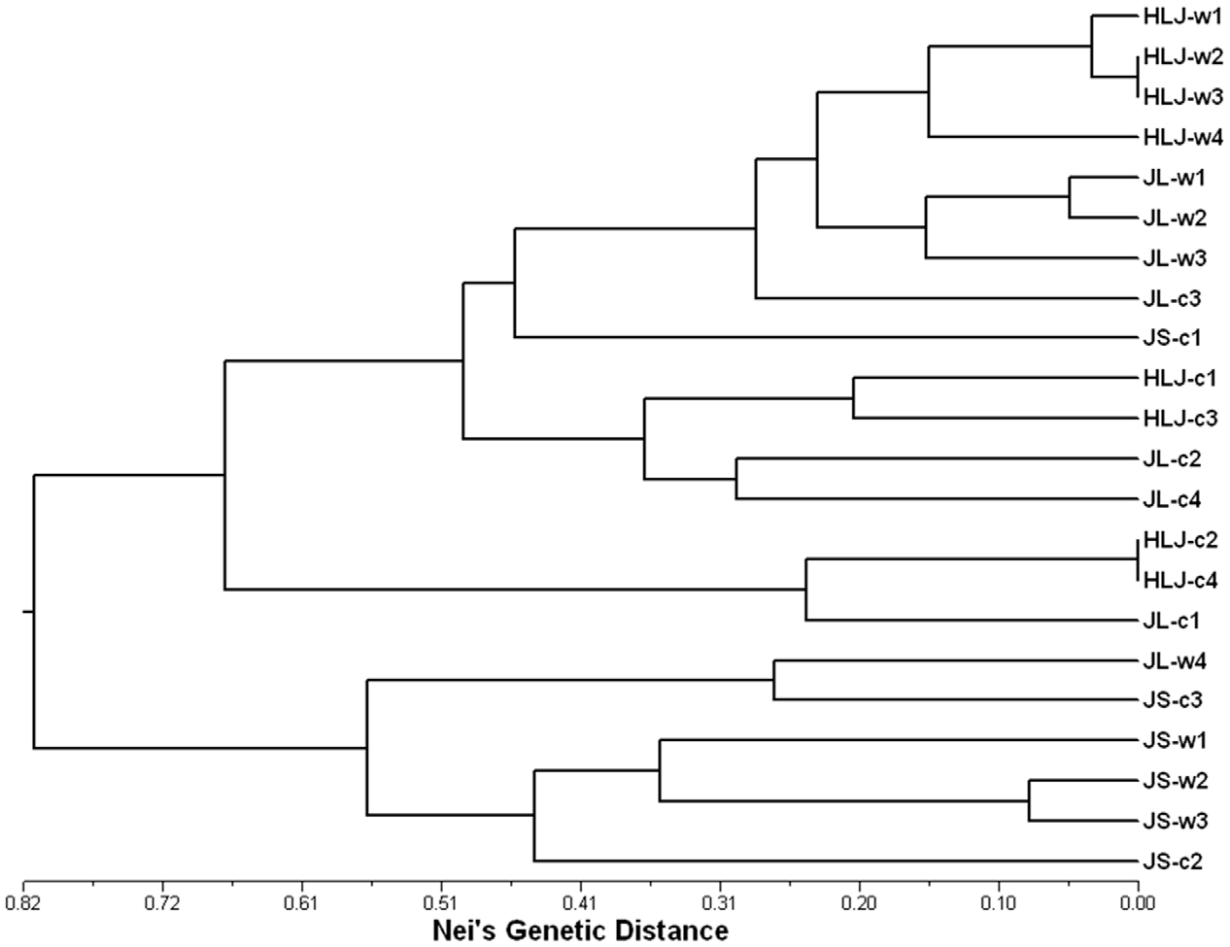
The UPGMA dendrogram of weedy rice populations and the coexisting rice varieties in the same fields, based on Nei's unbiased genetic distance of 4 shared SSR primer pairs (RM21, RM218, RM219, RM276) by the two taxa.

To examine the impacts of crop-weed introgression on genetic structure of weedy rice across a geographical range, genetic structure of weedy rice populations and the coexisting cultivated rice from the 11 locations was compared. The UPGMA tree of the 11 weedy rice populations generated based on the four shared SSR primer pairs was essentially the same as that based on 22 SSR primer pairs (data not shown). When the 11 coexisting rice cultivars were included in the cluster analysis, the structure of weedy rice populations indicated by the UPGMA tree was slightly altered (Fig. 5). In general, most weedy populations and the coexisting rice cultivars from the same regions are clustered together. There were exceptions in which a weed population (JL-w4) was clustered in the other group, and that a cultivar from Jiangsu (JS-c1) was clustered in the HLJ/JL group, indicating the possible influence on weedy-rice structure from introgression of rice cultivars.

## Discussion

### Outcrossing rate of weedy rice populations and its impacts on heterozygosity

Results from the present study based on >200 families (individuals) and >5500 progeny (seeds) indicated a low to moderate level of outcrossing rates (0.4∼11.7%) of weedy rice populations, although with considerable variation. The *t*-test of paired samples demonstrated that the observed inbreeding coefficients (*f_M_*) were in significant accordance with the expected inbreeding coefficients (*f_eq_*) in all 11 weedy rice populations. This statistic showed strong reliability of the estimated outcrossing rates of the self-pollination weedy rice based on the SSR fingerprints and MLTR modeling in this study. Therefore, the detected variation in outcrossing rates among weedy rice populations may well reflect the true nature of outcrossing in weedy rice, with a low possibility of errors from the experimental procedures.

Outcrossing rate is usually determined by the genetic ability to outcross (which is a relatively stable character for a particular plant population or species) and the environmental conditions under which the outcrossing events occur [12], [14], [15]. Similar to our finding, highly variable outcrossing rates (ca. 7∼51%) among weedy rice populations determined based on morphological traits and isozyme patterns were reported from previous studies [24], [47], although no explanation was made concerning the biological and environmental reasons that caused such a great variation. Biological factors may have played an essential role in determining outcrossing rates among weedy rice populations, although environmental conditions, such as air temperature, strength and direction of wind, and air humidity, could also considerably affect outcrossing rates. Previous studies indicated variable frequencies of gene flow from cultivated rice to diverse groups of weedy rice with different genetic backgrounds [20], [30], [31], [48], which is in a good accordance with data of outcrossing rates in this study. Our findings demonstrate that it is relatively easy for weedy rice to receive pollen from its sexually compatible rice cultivars (including GM rice) occurring at the close vicinity. More importantly, this study indicates the great variability of outcrossing rates among weedy rice populations in a relatively small geographical range, which is alarming for environmental biosafety assessment, as it suggests that the possibility of transgene escape from GM rice cannot be generalized from one field to another. The case-by-case principle should be rigidly followed in assessing the environmental impacts of transgene escape in rice.

In addition to variation in outcrossing rates, we also found that the proportion of biparental inbreeding (that denotes mating between individuals with identical or extremely similar genotypes) to the total outcrossing was considerably variable among weedy rice populations (ranging from ca.11∼65%). Usually, biparental inbreeding is associated with the fine-scale spatial genetic structure, in which the neighboring individuals are genetically identical or extremely close [49]. Therefore, the amount of biparental inbreeding observed in this study could be due to crosses between genetically close-related weedy rice individuals. The detected substructure in some weedy rice populations from this study suggested a certain degree of fine-scale genetic structure that could promote biparental inbreeding in weedy rice populations. An alternative explanation for biparental inbreeding might be the crosses between weedy rice and its closely related crop individuals. Given a much larger portion of crop plants than that of weedy rice plants in the same field, and the considerable overlap of flowering time between the two conspecific taxa, weedy rice plants have a much higher chance to hybridize with the crop than other weedy rice plants, resulting frequent crop-weed hybridization.

Our results, based on the fingerprinting of 22 SSR loci from another independent set of weedy rice samples with >300 individuals from the 11 populations, demonstrated a considerable among-population variation in genetic diversity, as estimated by the average number of alleles per locus, the percentage of polymorphic loci, and Nei's genetic diversity. Interestingly, these results showed a significantly positive correlation between outcrossing rates and observed heterozygosity (*H_o_*) in the corresponding weedy rice populations, although no such a correlation between outcrossing rates and Nei's genetic diversity or average number of alleles per locus was observed. In other words, outcrossing can considerably promote heterozygosity in weedy rice populations. Wright [16] suggested on the basis of his theoretical studies that the most direct effect of outcrossing was increase in frequency of heterozygosity in populations. In addition, empirical studies also indicated that heterozygous genotypes have a greater selective advantage than homozygous genotypes in plant populations, particularly for inbreeding species [50]–[53]. Segregation of heterozygous genotypes at various loci could generate numerous genetic recombinants in a population [1], [8]. Therefore, outcrossing and increased heterozygosity will play an important role in maintaining genetic variability in plant populations, as suggested by Anderson and Stebbins [4], Stebbins [54], Barton [2], and Stewart *et al*. [3]. Given the unique situation of weedy rice that occurs in rice fields and is accompanied by constantly changing new rice varieties, outcrossing with the crop can promote incorporation of new alleles to a weedy rice population, causing a genetic variation in the population.

### Introgression from cultivated rice possibly promoting weedy rice differentiation

Our results from the cluster analysis based on the coefficients of genetic distances showed evident differentiation among the 11 weedy rice populations. The Mantel test further indicated a significant correlation between the genetic and spatial distances of weedy rice populations across a geographical region (*r* ^2^ = 0.734; *P*<0.001) although the detected geographical effect might be associated with differences between north (Heilongjiang and Jilin) and south (Jiangsu) regions. Genetic differentiation of weedy rice populations has been reported by many authors [27], [55]–[57] with or without indication of association with their spatial distribution pattern. Gealy *et al*. [58] studied the genetic diversity and structure of weedy rice from USA, where geographic differentiation among weedy rice accessions across the country was found. However, the driving force that may cause genetic differentiation in weedy rice have rarely been discussed or explored. This will limit our profound understanding on how genetic differentiation in weedy rice has been developed. Particularly, for such a conspecific weedy taxon that coexists in crop fields and has a close contact with its coexisting crop, the identification of driving forces that can influence genetic differentiation of weedy rice populations is important.

Commonly, the causes of genetic differentiation among plant populations across a given geographical range can be associated with factors such as immigration/migration and environmental heterogeneity among populations [59]. Which of the factors have played a more important role in genetic differentiation of weedy rice populations? Similar to cultivated rice, weedy rice is a self-pollinating taxon with a high inbreeding coefficient (*F_is_*) [27], [56], [57], indicating its limited gene flow by pollination. In addition, seeds of weedy rice usually shatter to soil seed-banks after mature, showing restricted seed dispersal [22], [23]. Limited gene flow in general could contribute to the differentiation among weedy rice populations across a given geographical range if the environmental conditions are considerably different. However, weedy rice occurs in cultivated rice fields, of which the environment may not vary significantly. In the present study, weedy rice populations were collected in *Japonica* rice fields from NE and E China, where the farming conditions for rice are very similar in the irrigated eco-system. Normally, rice was cultivated once a year or rotated with wheat or oil rape at the following seasons, but rice varieties used across the region can vary greatly. Given the frequent gene flow from cultivated rice to weedy rice [20], [21], [60], the introgression of crop alleles from different rice varieties may contribute to the differentiation of weedy populations at different sites although other factors such as selection and habitat heterogeneity may also play their roles.

As a conspecific weed of cultivated rice, individuals of weedy rice are commonly surrounded by densely populated crop in the field. Recurrent gene flow from a particular rice variety or different varieties grown at different years in the same fields can considerably shape the genetic composition of weedy rice populations, and enhance genetic differentiation among weedy rice populations by incorporating crop alleles from different varieties [20], [27]. The hypothesis of gene flow and introgression from cultivated rice causing genetic differentiation of weedy rice can be tested in this study using molecular markers (like SSRs) because the population- or variety-specific alleles are easily identified in cultivated and weedy rice by paternity analysis. Detecting gene flow and introgression from cultivated to weedy rice is useful to explain the genetic differentiation and evolution of weedy rice populations. In addition, understanding the role of crop-to-weed gene flow and introgression can also be useful to address weed problems and particularly the environmental biosafety related issues caused by crop-to-weed transgene flow.

Data from outcrossing rate estimation in this study revealed some paternal-specific SSR alleles (paternity) that was not detected in the genotypes of maternal plants (weedy rice families). This finding suggests that the pollen donors of many outcrossing (or hybridization) events were most likely from cultivated rice, given the relatively low density (∼10 plants/100 m^2^) of weedy rice plants that were surrounded by cultivated rice plants in the fields. More importantly, comparison between the paternal-specific alleles found in weedy rice and alleles detected in cultivated rice from the same fields demonstrated some paternal-specific alleles that were also detected in rice varieties. This finding confirms the allelic introgression from diverse rice cultivars to weedy rice, supporting our hypothesis of introgression from rice crop that may influence genetic structure and differentiation of weedy rice populations. In fact, farmers frequently change rice varieties between years for cultivation among regions, where considerable genetic diversity of rice varieties has been detected [61]. Our cluster analysis further demonstrated that most weedy populations and the coexisting rice cultivars from the same regions are clustered together. However, some weed populations (e.g., JL-w4) and cultivars (e.g., JS-c1) were clustered in groups from other regions, indicating the possible influence of introgression from rice cultivars on the genetic structure of weedy populations.

The above findings have implications for understanding adaptive evolution of weedy rice in human-influenced agro-ecosystems. Weedy rice always occurs sympatrically with cultivated rice, and genes from cultivated rice can be easily incorporated into the gene pool of weedy rice through recurrent crop-to-weed gene flow and introgression. This progress may promote the persistence and better adaptation of weedy rice to the human-influenced habitats [62]. Under selection, particularly artificial selection, the introgression of crop alleles from different rice varieties through time can increase the similarity of weedy rice to the coexisting rice varieties, which can well explain the mimicry of weedy rice to it coexisting cultivated rice. In addition, such a process can promote genetic differentiation among weedy rice populations across a geographical range. Therefore, the impacts of gene flow and introgression from cultivated rice on genetic differentiation and adaptive evolution of weedy rice could not be ignored. This knowledge is particularly important for assessing transgene escape to weedy rice and its biosafety consequences when a transgene with natural selective advantages is possibly added and spread to the gene pool of weedy rice.

In summary, results from this study demonstrate a considerable variation in outcrossing rates and genetic diversity among weedy rice populations from a given geographical range. The correlation between outcrossing rates and observed heterozygosity provides evidence that a high level of outcrossing may influence genetic variability and potential evolution of weedy rice populations. This might be true for other self-pollinating plant species. Our results also show considerable genetic differentiation among weedy rice populations across a given geographical range. Noticeably, the genetic structure and differentiation of weedy rice populations could be affected by cultivated rice *via* gene flow and introgression although other factors such as selection and habitat heterogeneity may also play their roles. The knowledge of biological bases and the relationships among outcrossing, genetic diversity, and differentiation in weedy populations is essential for addressing adaptive evolution of weedy rice, in addition to assessing environmental biosafety impacts caused by crop-to-weed transgene flow from GM rice.
